# Incidence of Hepatocellular Carcinoma after Treatment with Sofosbuvir-Based or Sofosbuvir-Free Regimens in Patients with Chronic Hepatitis C

**DOI:** 10.3390/cancers12092602

**Published:** 2020-09-11

**Authors:** Eiichi Ogawa, Hideyuki Nomura, Makoto Nakamuta, Norihiro Furusyo, Eiji Kajiwara, Kazufumi Dohmen, Akira Kawano, Aritsune Ooho, Koichi Azuma, Kazuhiro Takahashi, Takeaki Satoh, Toshimasa Koyanagi, Yasunori Ichiki, Masami Kuniyoshi, Kimihiko Yanagita, Hiromasa Amagase, Chie Morita, Rie Sugimoto, Masaki Kato, Shinji Shimoda, Jun Hayashi

**Affiliations:** 1 Department of General Internal Medicine, Kyushu University Hospital, Fukuoka 8128582, Japan; furusyo@gim.med.kyushu-u.ac.jp; 2 The Center for Liver Disease, Shin-Kokura Hospital, Kitakyushu 8038505, Japan; hinomura55@haradoi-hospital.com; 3 Department of Internal Medicine, Haradoi Hospital, Fukuoka 8138588, Japan; 4 Department of Gastroenterology, National Hospital Organization Kyushu Medical Center, Fukuoka 8108563, Japan; nakamuta.makoto.ya@mail.hosp.go.jp; 5 General Internal Medicine, Taihaku Avenue Clinic, Fukuoka 8120039, Japan; 6 Kajiwara Clinic, Kitakyushu 8050019, Japan; kan@kajiwara-cl.jp; 7 Department of Internal Medicine, Chihaya Hospital, Fukuoka 8138501, Japan; dohmen-kzfm@cool.odn.ne.jp; 8 Department of Medicine, Kitakyushu Municipal Medical Center, Kitakyushu 8028561, Japan; k-akira1971july@jcom.home.ne.jp; 9 Department of Hepatology, Steel Memorial Yawata Hospital, Kitakyushu 8058508, Japan; ooho.a@ns.yawata-mhp.or.jp; 10 Department of Medicine, Kyushu Central Hospital, Fukuoka 8158588, Japan; heracles_azuma@kyushu-ctr-hsp.com; 11 Department of Medicine, Hamanomachi Hospital, Fukuoka 8108539, Japan; takahashi-k@hamanomachi.jp; 12 Center for Liver Disease, National Hospital Organization Kokura Medical Center, Kitakyushu 8028533, Japan; sato.takeaki.ky@mail.hosp.go.jp; 13 Department of Medicine, Fukuoka City Hospital, Higashi-ku, Fukuoka 8120046, Japan; koyanagi.t@fcho.jp; 14 Department of Internal Medicine, JCHO Kyushu Hospital, Kitakyushu 8068501, Japan; yichiki-gi@umin.ac.jp; 15 Department of Gastroenterology, Kyushu Rosai Hospital, Kitakyushu 8000296, Japan; kuniyoshi.intm-k@kyushuh.johas.go.jp; 16 Department of Internal Medicine, Saiseikai Karatsu Hospital, Karatsu 8470852, Japan; k_yanagt@po.saganet.ne.jp; 17 Amagase Clinic, Kitakyushu 8020065, Japan; dk-capricon-j-3@feel.ocn.ne.jp; 18 Department of Internal Medicine, Kyushu Railway Memorial Hospital, Kitakyushu 8000031, Japan; catirom39@pure.ocn.ne.jp; 19 Department of Gastroenterology, Kyushu Cancer Center, Fukuoka 8111395, Japan; sugirie5@rr.iij4u.or.jp; 20 Department of Medicine and Bioregulatory Science, Graduate School of Medical Sciences, Kyushu University, Fukuoka 8128582, Japan; mkato11@intmed3.med.kyushu-u.ac.jp; 21 Department of Medicine and Biosystemic Science, Graduate School of Medical Sciences, Kyushu University, Fukuoka 8128582, Japan; sshimoda@intmed1.med.kyushu-u.ac.jp; 22 Kyushu General Internal Medicine Center, Haradoi Hospital, Fukuoka 8138588, Japan; hayashij1949@haradoi-hospital.com

**Keywords:** hepatitis C virus, hepatocellular carcinoma, direct-acting antiviral, sofosbuvir

## Abstract

**Simple Summary:**

The development of hepatocellular carcinoma (HCC) has not been well-controlled, even after the elimination of hepatitis C virus (HCV), especially for those with cirrhosis or of high-age. Fibrosis-related biomarkers have been recognized as useful predictors for the development of HCC; however, there are few analyses of the HCC incidence for the various regimens with direct-acting antivirals (DAA). We found that DAA treatment with sofosbuvir, an oral nucleotide analogue inhibitor of HCV NS5B polymerase, was not associated with the development of de novo HCC within five years by propensity score matched analysis. Moreover, the distribution of the early stage of HCC (the Barcelona Clinic Liver Cancer stage 0/A) was similar for all treatment regimens, irrespective of the use of sofosbuvir.

**Abstract:**

Advanced fibrosis/cirrhosis and related biomarkers have been recognized as useful predictors of the development of hepatocellular carcinoma (HCC) by patients with chronic hepatitis C (CHC) following hepatitis C virus (HCV) cure by direct-acting antivirals (DAAs). However, it remains unclear if DAAs themselves have an influence on or facilitate the development of HCC. This multicenter cohort study included CHC patients without a history of HCC who achieved HCV elimination by DAAs. Cohorts of 835 patients treated with a sofosbuvir (SOF)-based regimen and 835 treated with a SOF-free regimen were matched 1:1 by propensity scoring with nine variables to evaluate differences in HCC incidence. The median observation period was 3.5 years. Sixty-nine cases of HCC were found during 5483.9 person-years (PY) over the entire follow-up period. The annual incidence was similar for both groups (SOF-based 1.25 and SOF-free 1.27 per 100 PY, respectively: adjusted hazard ratio (HR) 1.26, 95% confidence interval (CI) 0.75–2.12, *p* = 0.39). However, the annual incidence within the first two years was higher for patients treated with SOF than for those without, but did not reach significance (1.50 and 0.97 per 100 PY incidence rates, respectively: adjusted HR 2.05, 95% CI 0.98–4.25, *p* = 0.06). In summary, DAA treatment with SOF was not associated with an increase in the development of de novo HCC.

## 1. Introduction

Hepatitis C virus (HCV) was historically one of the leading causes of hepatocellular carcinoma (HCC) and liver-related mortality. Fortunately, with the advent of direct-acting antivirals (DAAs) in 2013, the rates of sustained viral response (SVR) now range from 85% to almost 100%, depending on the HCV genotype and the presence of cirrhosis or active HCC [1,2,3]. This has contributed to a reduction of the risk for HCC [4,5,6,7,8] and the number of patients requiring a liver transplant [9,10].

However, the development of HCC has not been well-controlled, even after HCV cure, especially for those with cirrhosis or in old age [4,5,6,7,8,11]. Some fibrosis-related biomarkers, such as FIB-4 index, aspartate aminotransferase to platelet ratio index, liver stiffness measurement, and α-fetoprotein (AFP) level, have been recognized as useful predictors for the development of HCC [4,5,11,12,13]. The underlying mechanisms of HCV-induced hepatocarcinogenesis have not been fully elucidated, and no clear evidence supports a tumorigenic role of DAAs. Molecular studies have indicated the potential effect of DAA treatment on angiogenesis [14,15] as potentially favoring HCC incidence. The results of a study with a prospective database showed no evidence for differential HCC incidence or recurrence risk following SVR from DAA and interferon-based therapy [16,17].

Sofosbuvir (SOF), an oral nucleotide analogue inhibitor of HCV NS5B polymerase, presents broad activity across genotypes and has a high barrier to resistance [18], which makes SOF the most promising drug of this class of DAAs. Nevertheless, SOF may have an impact on pathological processes in the liver via the induction of epidermal growth factor receptor (EGFR) signaling [19]. Many studies have found that the risk of HCC is reduced after SVR, but there are few analyses of the development of HCC for the various DAA regimens. This propensity score matched (PSM) analysis was carried out to assess the relative risk for the development of de novo HCC after SOF-based and SOF-free regimens.

## 2. Results

### 2.1. Baseline Demographics

**Figure 1:**
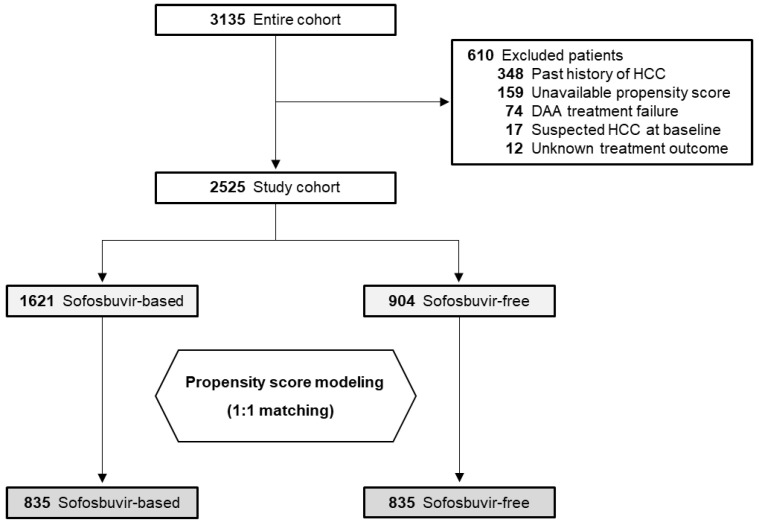
Study flowchart. HCC, hepatocellular carcinoma; DAA, direct-acting antiviral.

The study flowchart is shown in Figure 1. Among the entire cohort, 610 (19.5%) were excluded in accordance with the additional criteria, leaving the data of 2525 patients available for analysis. PSM identified 835 matched pairs, with no significant demographic differences except for HCV genotype, as shown in Table 1. The median observation period was 3.5 years (range: 0.3–5.5 years). Of the patients, approximately 43% were men, 26% had a diagnosis of cirrhosis, and 74% were treatment-naïve.

### 2.2. Cumulative Rates of the Development of HCC

**Figure 2:**
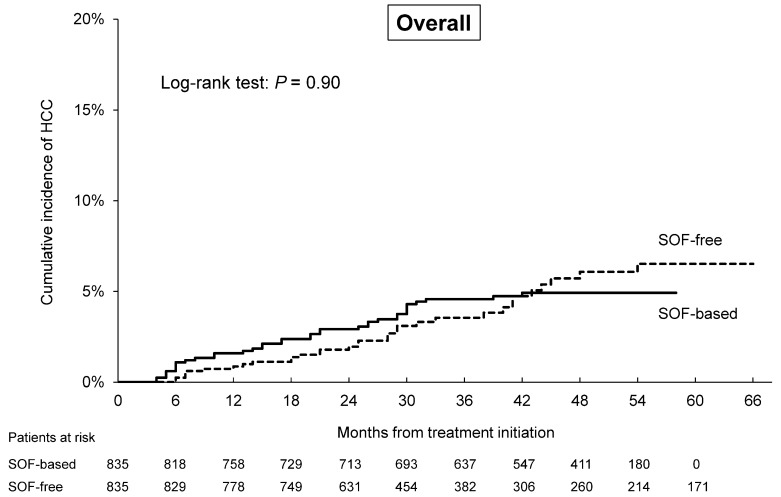
Cumulative rates of the development of hepatocellular carcinoma (HCC) in patients treated with DAA regimens with and without sofosbuvir (SOF). SOF-based: continuous line, SOF-free: dashed line.

**Figure 3:**
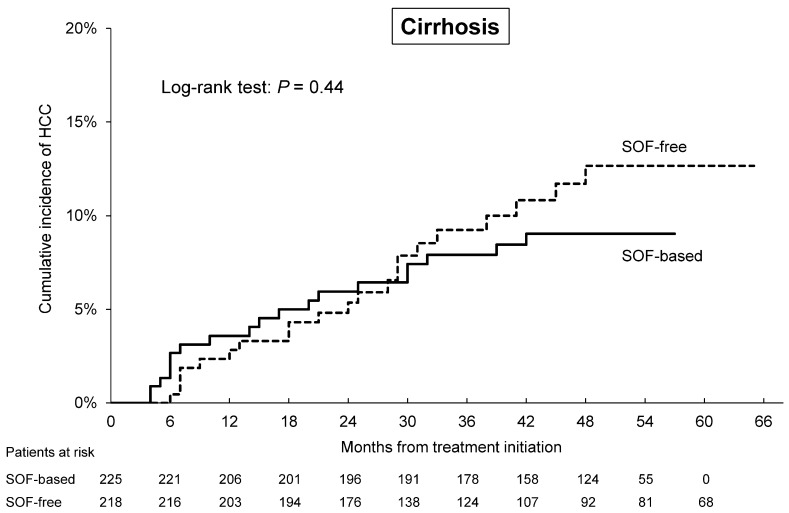
Cumulative rates of the development of hepatocellular carcinoma (HCC) in cirrhotic patients treated with DAA regimens with and without sofosbuvir (SOF). SOF-based: continuous line, SOF-free: dashed line.

The overall HCC incidence was 4.4% (*n* = 37) for patients treated with an SOF-based regimen and 3.8% (*n* = 32) for those treated with an SOF-free regimen. By Kaplan–Meier method, the cumulative rates of HCC incidence at two and four years were 3.5% and 4.9%, respectively, for patients treated with SOF and 2.0% and 6.1% for those SOF-free (*p* = 0.90 by log-rank test; Figure 2). For patients with cirrhosis, the cumulative rates of HCC incidence at two and four years were 6.0% and 9.0%, respectively, for patients treated with SOF and 5.4% and 12.7% for those SOF-free (*p* = 0.44 by log-rank test; Figure 3).

### 2.3. Incidence Rates for the Development of HCC within the First Two Years

There were 38 cases of HCC during 3074.2 person-years (PY) of follow-up within the first two years after treatment initiation. In our analysis of the incidence of HCC according to the regimen used, the annual incidence of HCC was higher for patients treated with SOF than for those without, but did not reach significance (1.50 and 0.97 per 100 PY incidence rates, respectively: adjusted hazard ratio (HR) 2.05, 95% confidence interval (CI) 0.98–4.25, *p* = 0.06) (Table 2, upper panel). For patients treated with SOF, there was no significant difference in the annual incidence of HCC between ribavirin and ribavirin-free groups (1.12 and 1.76 per 100 PY incidence rates, respectively: adjusted HR 0.67, 95% CI 0.25–1.81, *p* = 0.43) (Table 2, upper panel).

### 2.4. Incidence Rates for the Development of HCC over the Entire Follow-Up Period

There were 69 cases of HCC during 5483.9 PY over the entire follow-up period. Of the patients, 46 (66.7%) were stage 0/A HCC by the Barcelona Clinic Liver Cancer (BCLC) staging system, 15 (21.7%) were stage B, six (8.7%) were stage C, and two (2.9%) were stage D. There was no significant difference in the distribution of BCLC stage 0/A between the SOF-based and SOF-free groups (64.9% and 68.8%). The annual incidence of HCC was similar for both groups (SOF-based 1.25 and SOF-free 1.27 per 100 PY, respectively: adjusted HR 1.26, 95% CI 0.75–2.12, *p* = 0.39) (Table 2, lower panel). For patients treated with SOF, the annual incidence of HCC by univariate analysis was significantly higher for patients treated with ribavirin that for those ribavirin-free (1.64 and 0.74 per 100 PY, respectively: *p* = 0.046), probably because the SOF-based group had more elderly persons than the SOF-free group. No significant difference was found between the ribavirin-use and ribavirin-free groups by multivariable analysis (adjusted HR 0.56, 95% CI 0.24–1.29, *p* = 0.17) (Table 2, lower panel).

### 2.5. Predictors of HCC Development for the Overall Cohort

We analyzed the clinical parameters of patients who developed HCC and compared them with those who did not, as shown in Table 3. Univariate analysis extracted age > 70 (HR 2.30, *p* < 0.001), male sex (HR 1.67, *p* = 0.034), cirrhosis (HR 3.56, *p* < 0.001), treatment-experienced (HR 1.82, *p* = 0.016), serum albumin < 3.5 g/dL at 12 weeks after the end of treatment (pw12) (HR 2.86, *p* = 0.007), and AFP > 7 ng/mL at pw12 (HR 6.17, *p* < 0.001) as associated with HCC. There was no correlation between the development of HCC and body mass index, the presence of diabetes, alanine aminotransferase (ALT), or HCV RNA level.

Multivariable Cox regression analysis extracted age > 70 (adjusted HR 2.39, 95% CI 1.41–4.06, *p* = 0.001), male sex (adjusted HR 2.07, 95% CI 1.23–3.48, *p* = 0.006), cirrhosis (adjusted HR 2.97, 95% CI 1.78–4.92, *p* < 0.001), serum albumin < 3.5 g/dL at pw12 (adjusted HR 2.72, 95% CI 1.39–5.33, *p* = 0.003), and AFP > 7 ng/mL at pw12 (adjusted HR 4.92, 95% CI 2.72–8.88, *p* < 0.001) as independently associated with conferring higher risk for the development of HCC.

## 3. Discussion

Many reports have discussed the choice of the best-suited DAA regimen from the standpoint of fibrosis status, kidney function, treatment duration, or resistance-associated substitutions. In contrast, few reports have focused on the development of HCC. To the best of our knowledge, this study is the largest real-world study to evaluate HCC incidence after successful SOF-based and SOF-free treatment after adjusting for baseline characteristics. For our entire PSM cohort, we found similar rates of the development of HCC for patients cured of HCV by treatment with or without SOF. Moreover, the distribution of the early stage of HCC (the BCLC stage 0/A) was similar for all regimens, irrespective of the use of SOF. In our SOF-based and SOF-free groups, 4.9–6.1% developed HCC within four years (log-rank test: *p* = 0.90), and 9.0–12.7% of those with compensated cirrhosis developed HCC within four years (log-rank test: *p* = 0.44). The annual rate of HCC for the SOF-based group tended to be higher than that of the SOF-free group (1.5% vs. 0.9%) within two years after DAA initiation, but there was no significant difference (*p* = 0.06).

SOF is a pyrimidine nucleoside analogue that acts as an HCV RNA chain terminator by inhibiting HCV NS5B RNA polymerase, and can be used in combination with NS3/4A protease inhibitors, NS5A inhibitors, and/or ribavirin to achieve HCV elimination. SOF-based treatment has been shown to be highly effective and tolerable in both clinical trials and real-world data, irrespective of age, fibrosis status, or HCV genotype [20,21,22], although patients at an advanced CKD stage could be at risk of estimated glomerular filtration rate (eGFR) decline [23]. Our recent short-term analysis, within two years, consisted of patients treated with an SOF-based regimen and was done to determine predictive markers for HCC incidence [24]. In the present study, we sought to evaluate whether or not different DAA regimens have an influence on the development of HCC.

Several studies have implicated the role of overexpression and activation of EGFR in the progression of cirrhosis and the elevation of the EGFR level as being common to patients with HCC [25,26]. EGF may also combine with its receptor EGFR to initiate the downstream of phosphoinositide 3-kinase and extracellular regulated protein kinase signal pathway [27,28], and thus contribute to HCC proliferation, migration, and production of inflammatory cytokines, including C-X-C motif chemokine (CXCL)5 and CXCL8 [29]. Thus, EGF may act as an initiator factor to facilitate the transforming process of tumor cells from low metastatic potential into high metastatic potential, especially by acting on the metabolism of HCC cells [30,31]. In addition, an increase of other factors, such as vascular endothelial growth factor and angiopoietin-2, are potentially associated with the development of HCC [14,15]. In a recent report, SOF treatment was shown to activate EGFR-dependent signaling pathways, which were not observed with two other DAAs, the NS3/4A protease inhibitor simeprevir, or the NS5A inhibitor daclatasvir [19]. Moreover, a recent cohort study suggested that early HCC occurrence is strongly related to an SOF-based regimen without ribavirin [32]. We strongly agree that inflammatory cytokine or angiogenesis factors are associated with the development of HCC. Although molecular analysis was not done in this study, we were able to show such a tendency within two years after DAA initiation; however, the confirmed difference in the development of HCC between our SOF-based and SOF-free groups could not be proven. Moreover, the use of ribavirin was not extracted as a predictor of HCC incidence in the multivariable analysis of our cohort, which consisted of only patients without prior history of HCC treatment. Future studies regarding HCC recurrence in SOF-based and SOF-free groups will be needed to generalize our findings.

Large studies have shown a reduction in the risk of the development of HCC in the medium term, within five years. Comparative studies of DAA regimens and interferon-based therapies have not provided solid data; however, treatment with DAAs does not appear to be less effective than interferon-based regimens in reducing the development of HCC. One of the strengths of our study was that all patients continued surveillance during treatment and follow-up regardless of age and fibrosis status; therefore, it was possible to establish the appropriate timing of our diagnosis of HCC. In fact, only a few patients without cirrhosis developed HCC during the follow-up period, although the etiology of the development of HCC is unclear. Second, detailed laboratory data allowed us to differentiate the risk of HCC incidence, showing that age > 70, male sex, cirrhosis, treatment-experienced, serum albumin < 3.5 g/dL at pw12, and AFP > 7 ng/mL at pw12 were significantly associated with the development of HCC. Our results add to and expand the current knowledge of the incidence of HCC for patients who achieve HCV cure. Although SOF and ribavirin-based treatment were not significantly associated with the development of HCC, we advocate for careful assessment of cirrhosis and informed discussion with patients regarding their future HCC risk following HCV elimination.

We must acknowledge the limitations of our study. The first is that it was retrospective; however, there was little missing follow-up data, and what was missing could be inferred from the data we had available for use in our analyses because we enrolled consecutive patients treated with DAA in each hospital and reviewed every medical chart individually and in detail, including laboratory results and radiology reports. Second, there may have been selection bias for the DAA regimens because they were selected by the attending physician based on fibrosis status, kidney function, and HCV resistance-associated substitutions. This potential bias was ameliorated by our PSM analysis to control for a large number of patient characteristics, although the risk of unmeasured confounding cannot be ruled out.

## 4. Patients and Methods

### 4.1. Study Cohort

The Kyushu University Liver Disease Study (KULDS) Group consists of hepatologists from Kyushu University Hospital and its affiliated hospitals located in the northern Kyushu area of Japan. This large-scale, multicenter cohort study analyzed the data of 3135 consecutive Japanese patients who were enrolled from September 2014 through March 2020 for treatment with interferon-free DAA regimens for chronic HCV infection. Exclusion criteria for our original cohort were (1) under age 18 at the initiation of treatment, (2) decompensated cirrhosis (Child-Pugh B or C), (3) concomitant human immunodeficiency virus or hepatitis B virus infection, (4) excessive active alcohol consumption defined as > 20 g/day for women and > 30 g/day for men, and (5) history of organ transplantation. Moreover, to focus the study, we excluded patients (1) with a past history of HCC, (2) with DAA treatment failure, (3) with unknown DAA treatment outcome, (4) with suspected HCC at baseline, or (5) with an unavailable propensity score due to insufficient data.

This study was done with the approval of the Ethics Committees of Kyushu University Hospital, and each study site was registered as a clinical study on the University Hospital Medical Information Network (ID 000027342). Data were acquired from patients’ medical records stored in a prospectively maintained database of all patients who have been treated with DAAs.

### 4.2. Study Assessments

Clinical parameters were measured by standard laboratory techniques at a commercial laboratory within the three months before DAA initiation (baseline), every four weeks during DAA treatment, then every 12–24 weeks after achieving SVR, which was categorized as undetectable HCV RNA (target not detected) at pw12. HCV RNA was measured using a real-time reverse transcriptase PCR assay (COBAS TaqMan HCV assay, Version 2.0) (Roche Molecular Diagnostics, Tokyo, Japan) that has a lower limit of quantitation of 15 IU/mL. Cirrhosis was determined by transient elastography (FibroScan^®^; Echosens, Paris, France) or the presence of clinical, histological, radiologic, endoscopic evidence of cirrhosis, and/or portal hypertension (nodular contour on imaging, splenomegaly, and presence of varices). Diabetes mellitus was determined by medical history or baseline laboratory data (hemoglobin A1c ≥ 6.5% or fasting plasma glucose ≥ 126 mg/dL). The eGFR was calculated with the following formulas [33]: for men, eGFR (mL/min/1.73 m^2^) = 194  ×  serum creatinine level (sCr)^−1.094^  ×  age^−0.287^, and for women, eGFR = 194  ×  SCr^−1.094^  ×  age^−0.287^  ×  0.739.

The primary endpoint was the development of HCC after achieving SVR through SOF-based or SOF-free DAA regimens. The follow-up period reflects the time between the start date of DAA treatment and the date of the last image assessment. All patients were examined for HCC by abdominal ultrasonography, dynamic computed tomography, and/or magnetic resonance imaging at baseline, and every 3–6 months after the initiation of DAA treatment. The BCLC staging system was used for the classification of HCC [34].

### 4.3. Statistical Analysis

Standard descriptive and comparative statistics were done for all demographic and clinical variables. Categorical variables are described using proportions (%), and continuous variables are described as median (first-third quartile). We calculated the HCC incidence rate with 95% CI as the number of cases of HCC divided by total PY of follow-up. The Kaplan–Meier method was used to estimate the cumulative risk of HCC. The log-rank test was used to compare the difference in HCC incidence between the groups. We chose to use the PSM FUZZY extension command in Python to balance the SOF-based and SOF-free groups. Variables used in the PSM model included sex, age, serum albumin, ALT, platelet count, AFP, eGFR, cirrhosis, and treatment experience, all of which are clinically important variables for predicting the development of HCC according to recent reports [4,5,6,7,8,11,12,13,16,17]. Caliper matching of the propensity scores was done, and pairs were matched to within a range of 0.2 standard deviations of the logit of the propensity scores [35]. We assessed the adequacy of the propensity score specification by comparing the standardized difference in baseline covariates after stratification. A standardized mean difference of <0.1 was used to indicate no significant difference between baseline covariates. Cox proportional hazards regression was used to estimate the HR and 95% CI for the risk of developing HCC during the follow-up period.

All statistical analyses were conducted using SPSS Statistics version 25.0 (IBM SPSS Inc., Chicago, IL, USA). Statistical significance was defined using a two-tailed *p*-value < 0.05.

## 5. Conclusions

In this large, multicenter analysis, DAA treatment with SOF was not associated with the development of de novo HCC, after adjusting for sex, age, serum albumin, ALT, platelet count, AFP, eGFR, cirrhosis, and treatment experience. We believe our study provides further insight into the development of HCC for specific DAA regimens. However, further detailed analysis within a short period after SVR will be necessary to confirm the effect of SOF on de novo HCC and HCC recurrence.

## Figures and Tables

**Table 1:** Baseline characteristics after propensity score matching.

Characteristics	Sofosbuvir-Based (*n* = 835)	Sofosbuvir-Free (*n* = 835)	Standardized Mean Difference *
**Age**	67 (56–75)	67 (57–74)	0.04
**Male**	365 (43.7)	360 (43.1)	0.02
**Body Mass Index (kg/m^2^)**	22.9 (20.6–25.3)	22.7 (20.6–25.0)	0.11
**Cirrhosis**	225 (26.9)	218 (26.1)	0.02
**Diabetes**	151 (18.2)	129 (15.4)	0.10
**Treatment-Naïve**	613 (73.4)	621 (74.4)	0.03
**Total Bilirubin (mg/dL)**	0.7 (0.6–0.9)	0.7 (0.5–0.9)	0.09
**Albumin (g/dL)**	4.1 (3.8–4.3)	4.1 (3.8–4.3)	0.03
**AST (U/L)**	42 (29–62)	42 (29–63)	0.08
**ALT (U/L)**	40 (26–63)	39 (25–66)	0.07
**γGTP (U/L)**	32 (20–58)	32 (20–57)	0.02
**eGFR (mL/min/1.73 m^2^)**	72 (62–84)	74 (62–86)	0.01
**Platelet count (10^3^/** **μ** **L)**	159 (117–205)	161 (118–205)	0.05
**α-fetoprotein (ng/mL)**	4.5 (2.9–8.0)	4.4 (2.8–8.3)	0.03
**HCV RNA (logIU/mL)**	6.1 (5.5–6.5)	6.1 (5.6–6.5)	0.14
**HCV Genotype**			
**Genotype 1**	497 (59.5)	705 (84.4)	
**Genotype 2**	338 (40.5)	130 (15.6)	0.82
**Other Genotypes**	0	0	
**DAA regimen**			
**LDV/SOF**	500 (59.9)	-	
**SOF + RBV**	335 (40.1)	-	
**ASV + DCV**	-	314 (37.6)	
**EBR + GZR**	-	227 (27.2)	
**GLE/PIB**	-	216 (25.9)	
**2D**	-	78 (9.3)	

Data are expressed as median (first-third quartiles) or number (%). AST, aspartate aminotransferase; ALT, alanine aminotransferase; γGTP, gamma-glutamyl transpeptidase; eGFR, estimated glomerular filtration rate; HCV, hepatitis C virus; DAA, direct-acting antiviral; LDV, ledipasvir; SOF, sofosbuvir; RBV, ribavirin; ASV, asunaprevir; DCV, daclatasvir; EBR, elbasvir; GZR, grazoprevir; GLE, glecaprevir; PIB, pibrentasvir; 2D, ombitasvir/paritaprevir/ritonavir. * Values < 0.1 indicate adequate balance between the SOF-based and SOF-free groups.

**Table 2:** Incidence rate of hepatocellular carcinoma, stratified by the use of sofosbuvir or ribavirin.

Treatment	Patients, *n*	HCC, *n*	PY of Follow-Up *	Incidence of HCC per 100 PY (95% CI)	Crude HR (95% CI)	*p* Value	Adjusted HR ** (95% CI)	*p* Value
**Within the First Two Years**								
**Sofosbuvir**								
**- Free**	835	15	1539.4	0.97 (0.58–1.62)	1	0.19	1	0.06
**- Based**	835	23	1534.8	1.50 (0.99–2.25)	1.55 (0.81–2.96)		2.05 (0.98–4.25)	
Ribavirin ***								
**- Free**	500	16	909.8	1.76 (1.06–2.86)	1	0.33	1	0.43
**- Based**	335	7	625.1	1.12 (0.49–2.34)	0.64 (0.26–1.56)		0.67 (0.25–1.81)	
**Entire Follow-Up Period**								
**Sofosbuvir**								
**- Free**	835	32	2565.1	1.25 (0.88–1.76)	1	0.90	1	0.39
**- Based**	835	37	2918.8	1.27 (0.92–1.75)	0.97 (0.60–1.56)		1.26 (0.75–2.12)	
Ribavirin ***								
**- Free**	500	28	1710.3	1.64 (1.13–2.37)	1	0.046	1	0.17
**- Based**	335	9	1208.4	0.74 (0.37–1.43)	0.47 (0.22–0.99)		0.56 (0.24–1.29)	

HCC, hepatocellular carcinoma; PY, person-years; HR, hazard ratio; CI, confidence interval. * PY of follow-up was calculated from treatment initiation. ** Adjusted for sex, age, diabetes, cirrhosis, HCV RNA, treatment experience, serum albumin, and alpha-fetoprotein at pw12. *** Comparison with sofosbuvir/ledipasvir and sofosbuvir/ribavirin groups.

**Table 3:** Predictors of hepatocellular carcinoma development in the propensity score matched cohort.

Characteristics	Univariable Analysis		Multivariable Analysis	
	HR (95% CI)	*p*-Value	Adjusted HR (95% CI)	*p*-Value
Age				
<70	1 (Referent)	<0.001	1 (Referent)	0.001
≥70	2.30 (1.42–3.73)		2.39 (1.41–4.06)	
Sex				
Female	1 (Referent)	0.034	1 (Referent)	0.006
Male	1.67 (1.04–2.68)		2.07 (1.23–3.48)	
Body Mass Index (kg/m^2^)				
<25	1 (Referent)	0.85		
≥25	0.95 (0.55–1.64)			
Fibrosis Status	1 (Referent)		1 (Referent)	
Non-Cirrhosis	3.56 (2.21–5.79)	<0.001	2.97 (1.78–4.92)	<0.001
Cirrhosis				
Diabetes	1.08 (0.57–2.06)	0.82		
History of HCV Treatment				
Treatment-Naïve	1 (Referent)	0.016	1 (Referent)	0.057
Treatment-Experienced	1.82 (1.13–2.94)		1.65 (0.97–2.80)	
HCV RNA (log_10_ IU/mL)				
<6.0	1 (Referent)	0.43		
≥6.0	1.22 (0.75–1.99)			
Serum Albumin (g/dL) at pw12				
>3.5	1 (Referent)	0.007	1 (Referent)	0.003
≤3.5	2.86 (1.46–5.58)		2.72 (1.39–5.33)	
ALT (U/L) at pw12				
<30	1 (Referent)	0.078	1 (Referent)	0.57
≥30	1.70 (0.94–3.05)		1.20 (0.64–2.27)	
α-Fetoprotein (ng/mL) at pw12				
≤7	1 (Referent)	< 0.001	1 (Referent)	<0.001
>7	6.17 (3.72–10.2)		4.92 (2.72–8.88)	
DAA Regimen				
Sofosbuvir-Based	1 (Referent)	0.90		
Sofosbuvir-Free	0.97 (0.60–1.56)			

HCV, hepatitis C virus; pw12, 12 weeks after the end of treatment; ALT, alanine aminotransferase; DAA, direct-acting antiviral; HR, hazard ratio; CI, confidence interval.

## Abbreviations

HCV	hepatitis C virus
HCC	hepatocellular carcinoma
DAA	direct-acting antiviral
SVR	sustained viral response
AFP	α-fetoprotein
SOF	sofosbuvir
EGFR	epidermal growth factor receptor
PSM	propensity score matching
pw12	12 weeks after the end of DAA treatment
eGFR	estimated glomerular filtration rate
sCr	serum creatinine
CI	confidence interval
PY	person-years
ALT	alanine aminotransferase
HR	hazard ratio
CXCL	C-X-C motif chemokine
